# Exogenous application of stress-related signaling molecules affect growth and cannabinoid accumulation in medical cannabis (*Cannabis sativa* L.)

**DOI:** 10.3389/fpls.2022.1082554

**Published:** 2022-12-20

**Authors:** José Garrido, Saleta Rico, Carolina Corral, Conchi Sánchez, Nieves Vidal, Juan José Martínez-Quesada, Carlos Ferreiro-Vera

**Affiliations:** ^1^ Phytoplant Research Sociedad de Responsabilidad Limitada Unipersonal (S.L.U), Departamento de Hibridación y Cultivo, Parque Científico-Tecnológico de Córdoba, Córdoba, Spain; ^2^ Departamento Fisiología Vegetal, Misión Biológica de Galicia (MBG)-Spanish Research Council (CSIC), Santiago de Compostela, Spain

**Keywords:** medical cannabis, elicitors, THCA, CBDA, salicylic acid, jasmonic acid, γ-aminobutyric acid

## Abstract

Medical cannabis (*Cannabis sativa* L.) is a source of bioactive phytochemicals with promising pharmacological and therapeutic applications. Enhancing the accumulation of valuable bioactive compounds is potentially a way of increasing the economic importance of this crop. Signaling molecules like salicylic acid (SA), jasmonic acid (JA), and γ-aminobutyric acid (GABA) are involved in the regulation of plant development and responses to biotic and abiotic stresses. Moreover, several phytohormones regulate plant trichome formation and elicit the synthesis of secondary metabolites in many plant species in both *in vitro* and *in vivo* systems. Therefore, exogenously delivered plant signaling molecules have the potential to modify the chemical profiles of medical cannabis. In this study, we found that the foliar application of SA, methyl jasmonate (MeJA), and GABA produces changes in the accumulation of the two major cannabinoids, cannabidiolic acid (CBDA) and Δ^9^- tetrahydrocannabinolic acid (THCA), in leaves and inflorescences of a medical cannabis variety. MeJA at 0.1 mM increased the CBDA content in inflorescences by 15.6%, while SA and MeJA at 0.1 mM increased CBDA and THCA accumulation in leaves by up to 57.3%. Treatments did not change the expression of genes participating in the final steps of the biosynthetic pathway of cannabinoids: *olivetolic acid cyclase* (*CsOAC-1* and *CsOAC-2*), *2-acylphloroglucinol 4-prenyltransferase* (*CsPT4*), *cannabidiolic acid synthase* (*CsCBDAS*), and *tetrahydrocannabinolic acid synthase* (*CsTHCAS*). Trichome density was not significantly different from the control plants in any treatment. Besides, we found strong correlations between several plant growth parameters and cannabinoid yields, showing a direct link between plant fitness and the production of cannabinoids.

## 1 Introduction

*Cannabis sativa* L. is an annual and predominantly dioecious species originating from Asia, with a long domestication history because of its many traditional uses, including textile fibre source, seeds for nutrition, and medicinal or recreational drug use (Andre et al., 2016; Ren et al., 2021). In modern times the interest in this multi-purpose plant has experienced a continuous expansion due to the increasing new applications in different areas, such as nutrition, food additives, and nutraceuticals (Ascrizzi et al., 2020; Rupasinghe et al., 2020); emerging green materials (Moscariello et al., 2021; Sorrentino, 2021); production of bioethanol and biofuels (Brar et al., 2022); or bioremediation and restoration of contaminated water and soils (Loffredo et al., 2021; Pudełko et al., 2021). However, the most economically attractive feature is the plant richness in different bioactive phytochemicals with promising pharmacological and therapeutic applications such as flavonoids, terpenes, and, particularly, phytocannabinoids (Andre et al., 2016). So far, the cannabis market has experienced continuous growth, and it is expected to reach up to 40.6 billion by 2024 (Brar et al., 2022).

Cannabis glandular trichomes are the specialized structures responsible for the synthesis and accumulation of essential oils, rich in terpenes and cannabinoids. They are classified in three types based on their morphologies: bulbous, sessile, and stalked. Trichomes are also different in their metabolic profiles (Livingston et al., 2020) and distribution in leaves and inflorescences (Turner et al., 1980). The stalked glandular trichomes accumulate more total cannabinoids than the other types. They are found exclusively on the surface of perigonal bracts and the small companion leaves (“sugar leaves”) of the female raceme inflorescences and develop from the sessile trichomes under short day photoperiod (Spitzer-Rimon et al., 2019; Livingston et al., 2020; Tanney et al., 2021). More than 113 different cannabinoids are produced by *C. sativa*, among which the most abundant are cannabigerolic acid (CBGA), cannabidiolic acid (CBDA), Δ^9^-tetrahydrocannabinolic acid (THCA), and cannabichromenic acid (CBCA), together with their decarboxylated neutral forms (CBG, CBD, THC, and CBC, respectively) (Gülck and Møller, 2020). Cannabinoid biosynthesis has been recently elucidated, comprising as final steps the synthesis of olivetolic acid by the enzyme olivetolic acid cyclase (CsAOC), which is prenylated by prenyl transferases (CsPT) to yield CBGA (Luo et al., 2019; Gülck and Møller, 2020). Depending on the genotype, CBGA is then used as a substrate by plant synthases (CsTHCAS, CsCBDAS, and CsCBCS) to produce these three major biosynthetic cannabinoids.

The biological roles commonly attributed to glandular trichomes and their resinous secretion are related to defensive adaptations to abiotic and biotic stresses caused by environmental conditions and herbivores, and are consistent with their sunscreen, insect repellent, toxic and antimicrobial activities (Tanney et al., 2021). Therefore, several attempts to use stress as means to manipulate resin accumulation in medical cannabis have been carried out (reviewed in Gorelick and Bernstein, 2017). In recent studies, the application of several abiotic (flooding, herbicide treatment, wounding damage, heat, and drought) and biotic (*Golovinomyces spadiceus, or Manduca sexta*) stresses has been evaluated in up to 4 medical cannabis varieties (Toth et al., 2021; Park et al., 2022). In their experimental setups, drought stress applied during the early flowering stage was effective to cause changes in the cannabinoids profiles, resulting in a significant accumulation of CBG in inflorescences, but up to 70-80% decrease in THC and CBD accumulation, whereas other treatments did not exert any beneficial effects on the cannabinoid accumulation (Park et al., 2022). These results contrast with the ones obtained in the “Nebula” strain, in which the application of drought stress approximately 5-6 weeks after the flowering stage initiation significatively increased yields of the major cannabinoids THCA/THC and CBDA/CBD (Caplan et al., 2019). On the other hand, the cannabis strain RCK23 reacted to biotic stress caused by the spider mite *Tetranychus urticae* by increasing the quantities of most terpenes and some cannabinoids in both leaves and flowers (Kostanda and Khatib, 2022).

Phytohormones are involved in the regulation of several plant adaptations to environmental changes and stresses, regulating plant defense, growth, and developmental responses through changes in gene expression and crosstalk (Rivas-San Vicente and Plasencia, 2011; Vos et al., 2015; Huang et al., 2017; Siddiqi and Husen, 2019; Li et al., 2022; Nguyen et al., 2022). Up to now, salicylic acid (SA) and jasmonic acid (JA) have been the most commonly studied, as they constitute the backbone of plant defense responses against microbial pathogens and wounding caused by herbivorous insects (Pieterse et al., 2012) and play a key role in abiotic stress responses (Santisree et al., 2020). Other phytohormones and signaling molecules that participate in the modulation of biotic and abiotic stress responses are ethylene (ET), cytokinins, auxins, gibberellic acid (GA_3_), brassinosteroids, strigolactones, abscisic acid (ABA), or the non-proteinogenic amino acid γ-Aminobutyric acid (GABA) (Verma et al., 2016; Bürger and Chory, 2019; Li et al., 2021b). Interestingly, several of the aforementioned compounds (e.g., SA and JA) are important regulators of trichome initiation and development (Li et al., 2021a), as well as elicitors in the synthesis of secondary metabolites of interest in many species, in both *in vitro* and *in vivo* systems (Jan et al., 2021). Therefore, stress-related signaling molecules have the potential to modify the chemical profile of medical cannabis and enhance the accumulation of valuable bioactive compounds.

So far, the effects of phytohormones and other signaling compounds in trichome development and the elicitation of synthesis and accumulation of cannabinoids in medical cannabis are poorly understood. In an *in vitro* elicitation study, the application of SA, JA, or the conjugated form Methyl-jasmonate (MeJA) to cell suspension cultures of cannabis didn’t produce changes in the expression of THCAS or detectable THC accumulation (Flores-Sanchez et al., 2009). Mansouri et al. investigated the effects of several phytohormones on seed-derived populations of cannabis plants and found that ABA produced a decrease in the accumulation of THC and CBD during the vegetative stage (Mansouri and Asrar, 2012), although they had reported an increase in THC content in leaves and inflorescences of male and female plants in a previous study (Mansouri et al., 2009b). The same authors also reported a decrease in the THC accumulation as a result of gibberellic acid (GA_3_) treatment (Mansouri et al., 2009a), whereas ethephon (an ET-releasing plant regulator) treatments increased THC content up to 9 fold in the vegetative stage and CBD showed a dose-dependent response (Mansouri et al., 2016). However, in other hemp varieties, ethephon treatment did not cause significant changes in cannabinoid accumulation or CBD : THC ratios (Toth et al., 2021). In another study, Jalali et al. performed treatments with SA and GABA in seed-derived plants of the drug-type “Saghez” variety, reporting changes in both enzyme expression of polyketide synthase (OLS), aromatic prenyltransferase (PT), Δ9-tetrahydrocannabinolic acid synthase (THCAS) and cannabidiolic acid synthase (CBDAS), as well as THC/CBD accumulation, depending on the elicitor concentration (Jalali et al., 2019).

The sometimes-contrasting results described above suggest that additional research is required to achieve the practical use of stresses or elicitation, to enhance the synthesis of bioactive compounds in multiple genotypes of medical cannabis. Therefore, our objective was to test the application of aqueous sprays of SA, MeJA, and GABA as a practical method to elicit cannabinoid synthesis in medical cannabis varieties. To the best of our knowledge, we have investigated the effects of these three signaling compounds on plant growth and glandular trichomes formation in Cannabis sativa L. for the first time. In addition, we describe dosage-dependent and differential effects in the cannabinoid accumulation in the leaves and inflorescences and explore the association between plant growth, trichome formation, and the resulting cannabinoid yields. Our results can be applied to improve the cultivation of medical cannabis varieties, enhancing plant growth and cannabinoid yields.

## 2 Material and methods

### 2.1 Plant material and culture conditions

All the work in this study was carried out under a production and research license for medical purposes, issued by the AEMPs (Ministerio de Sanidad, Spain) to Phytoplant Research S.L.U. A proprietary type II (Small and Beckstead, 1973) medical cannabis variety (“Beatriz”; App. No. 20170146; https://cpvo.europa.eu/en) with a CBD : THC ratio of 1:1 (total cannabinoids 16-22%) was chosen to conduct this study. Cuttings were taken from 3 months-old genetically identical female plants, growing under an 18/6 h light/dark cycle in a controlled environment growing room. Twenty-one days-old, rooted cuttings (10-11 cm tall and six leaves, on average) were transplanted in 3 L pots, containing a custom blend of CANNA Coco Professional Plus and perlite (60:40), then acclimated for two additional weeks before switching to a 12/12 h light/dark cycle for ten weeks to induce flowering. Plants were cultured at 25 ± 2 °C, 50-55% relative humidity, and supplied with 800 ppm CO_2_ and 395 ± 69 μmol m^-2^ s^-1^ at the canopy level, provided by 600W Metal Halide bulbs (Philips GreenPower Plus 600W EL E40). Rooted plants were fertilized with CANNA Coco following the manufacturer’s indications (https://www.cannagardening.com/growguide). A total of 99 female plants were used in this study, randomly divided into nine treatment groups (9 plants each) and a control group (18 plants) in growing benches (9 plants/m^2^).

### 2.2 Plant treatments

Methyl jasmonate, 95% (Sigma-Aldrich), γ-Aminobutyric acid (Sigma-Aldrich), and Salicylic acid (Duchefa Biochemie) were prepared at 0.1, 1, and 10 mM in distilled water with 0.01% (v/v) Tween 20 (Sigma-Aldrich) and sprayed on plant leaves and inflorescences to the point of runoff. Control plants (mock-treated) were sprayed with a 0.01% (v/v) Tween 20 solution. Treatment groups were separated from each other by polycarbonate screens during phytohormone applications to prevent cross-contamination. All plants were treated once per week during the generative stage (8 applications total) until the week before harvesting.

### 2.3 Sample collection

Samples of fan leaves, sugar leaves, and perigonal bracts were taken one week before harvesting from 3 plants per treatment, and glandular trichomes (sessile and stalked) were counted by examining sections ranging from 4 to 25 mm^2^ (depending on the source’s sample size) under a Motic SMZ171 stereo microscope. The stalked glandular trichomes density (number of trichomes per mm^2^) in inflorescences was assessed on the adaxial surface of perigonal bracts, and both adaxial and abaxial surfaces in the sugar leaves. Besides, we counted the number of sessile glandular trichomes on the adaxial surface of fan leaves from the same plants.

Plant height, number of inflorescences, and total fresh weight (FW) were recorded at the harvesting date. Plants were dried at 23 ± 1°C for 14 days until they reached 10-12% of the initial FW. Then, apical inflorescence dry weight (ADW), total inflorescences dry weight (FDW) and leaves dry weight (LDW) were measured. Inflorescence-accompanying leaves (“sugar leaves”) were not trimmed and therefore were counted as inflorescence biomass for the ADW and FDW determination. For the determination of cannabinoids, inflorescences, and leaves from every three plants (treatments), or six plants (control) were pooled to get three samples per treatment.

### 2.4 Analysis of cannabinoids

Inflorescences and leaves samples were analyzed by near infrared spectroscopy (NIRS) for the determination of the content of cannabinoids. A FOSS NIR DS2500 series (FOSS, Hillerød, Denmark, EU) was used for reflectance measurement of the samples from 400 to 2499.5 nm, every 0.5 nm, equipped with a Si detector for the 400-1100 nm region and a PbS detector for the 1100-2500 nm region. For sample measurement, a circular quartz capsule was employed. Spectral absorbance values were recorded in the reflectance mode as log 1/R, where R is the sample reflectance. ISIscan Nova software version 7.9.1.2 was used for spectra acquisition. Additionally, high performance liquid chromatography coupled to a diode array detector (HPLC-DAD) was employed as reference technique to assure the correct performance of NIRS measurements. An Agilent 1260 Infinity series (Agilent Technologies, Inc, Santa Clara, USA) equipped with a G1329B autosampler and a G1316A DAD was employed. For the chromatographic separation, a Restek Raptor ARC18 (150x2.1 mm, 1.8μm particle size, Restek Corporation, Bellefonte, Pennsylvania, USA) column was used. The separation of the compounds was carried out following an isocratic method at a flow rate of 0.37 mL/min. The mobile phase was composed of 73% B (acetonitrile with 0.1% (v/v) formic acid) and 27% A (milli-Q water with 5 mM ammonium formate and 0.1% formic acid) and the oven temperature was set at 35°C. The injected sample volume was 1 µL. Agilent LC OpenLAB software was employed for data treatment.

For HPLC analysis, approximately 0.5 g aliquots of dry plant material were weighed in 50 mL Falcon tubes and extracted with 5 mL of methanol in an ultrasound bath for 30 min. The supernatant was filtered through 0.22 µm syringe nylon filters and collected in 4 mL glass vials. Prior to HPLC-DAD analysis, proper dilutions of the samples were performed. For NIRS measurements, a circular quartz capsule was filled with plant material previously dried and grounded. Samples were gently pressed with a circular metal weight to homogenize the surface and avoid potential measurement interferences from gaps. The CBDA and THCA contents were calculated as mg of cannabinoid produced per plant, either from inflorescences or the leaf dry biomass.

### 2.5 RNA extraction, primers used and RT-qPCR

Two weeks before harvesting, floral buds (about 1 g) were collected from three random plants per treatment, 24 h after foliar spraying, and frozen in liquid nitrogen. Total RNA was extracted from 100 mg of frozen tissue using the IQ Easy Plus Plant RNA Extraction Mini Kit (iNtRON Biotechnology), following the manufacturer’s recommended protocol for leaf and seed tissues, followed by phenol-chloroform extractions. The RNA concentration and purity were determined spectrophotometrically in a Nanodrop 1000 (Thermo Fisher Scientific ™, USA), and RNA integrity was checked by visualization in a 1.2% (w/v) agarose gel. RNA samples were stored at -80°C until use.

First-strand cDNA was synthesized from 1μg of total RNA using the High-Capacity cDNA Reverse Transcription Kit (Applied Biosystems), according to the manufacturer’s instructions. The qPCR was performed in an optical 48-well plate with a Step One Real Time PCR System (Applied Biosystems, Foster City, CA, USA). PCR reactions contained 300-900 nM of each primer (**Supplementary Table 1**), 1 µl of cDNA template (8.33 ng of input RNA), and 2x Power SYBR^®^ Green PCR Master Mix (Applied Biosystems) in a final volume of 20 µl. The PCR thermal profile used was an initial step of 95° C/10 min, followed by 40 amplification cycles of 95° C/15 s, 60° C/1 min., and a final dissociation protocol to obtain the melting profiles. Three biological and three technical replicates were assessed for each sample. Relative expression values were expressed as fold-change using the comparative CT method (ΔΔCT Method) (Schmittgen and Livak, 2008). Results are expressed as relative values to floral buds of control plants, and as means ± standard error from three biological replicates.

Specific primer pairs for *CsTHCAS*, *CsCBDAS*, *CsPT4*, and two *CsOAC* were designed with Primer-BLAST (https://www.ncbi.nlm.nih.gov/tools/primer-blast/) using reference GeneBank sequences (**Supplementary Table 1**). Two *olivetolic acid cyclase* genes (*CsAOC-1* and *CsAOC-2*) and seven *prenyltransferase*-like genes (LOC115713185, LOC115707809, LOC115713148, LOC115713171, LOC115713205, LOC115713215, and LOC115722991) were identified in the CBDRx reference genome (GenBank GCF_900626175.2), but *CsPT4* (LOC115713185) was selected for this study as it was the only one found functional in transgenic yeast (Luo et al., 2019). Amplification of three reference genes, *Elongation factor 1 alpha*, *Serine/threonine-protein phosphatase* and *Tubulin α -1* (Guo et al., 2018; Deguchi et al., 2021), selected based on their stability in *C. sativa*, was used as an internal control for normalizing the relative expression data. Primer efficiencies were tested by comparison against a standard curve for each gene.

### 2.6 Statistical analyses

Statistical analyses were performed by Statistix 10. Levene´s test was used to verify the homogeneity of variances. Data were analyzed for statistical significance by one-way ANOVA, followed by Dunnett’s test (two-tailed) for multiple comparisons with the control treatment, or by Kruskal-Wallis (K-W) and Dunn’s test. Differences between the results were considered significant at P≤ 0.05 or 0.1. Pearson’s correlation was used to assess relationships between different growth parameters and the production of cannabinoids (**Supplementary Table 2**). A correlogram was generated using the R corrplot package (https://github.com/taiyun/corrplot).

## 3 Results

### 3.1 Effects on plant growth, inflorescence formation, and glandular trichomes density

#### 3.1.1 Height increment, fresh and dry weight

**Figure 1:**
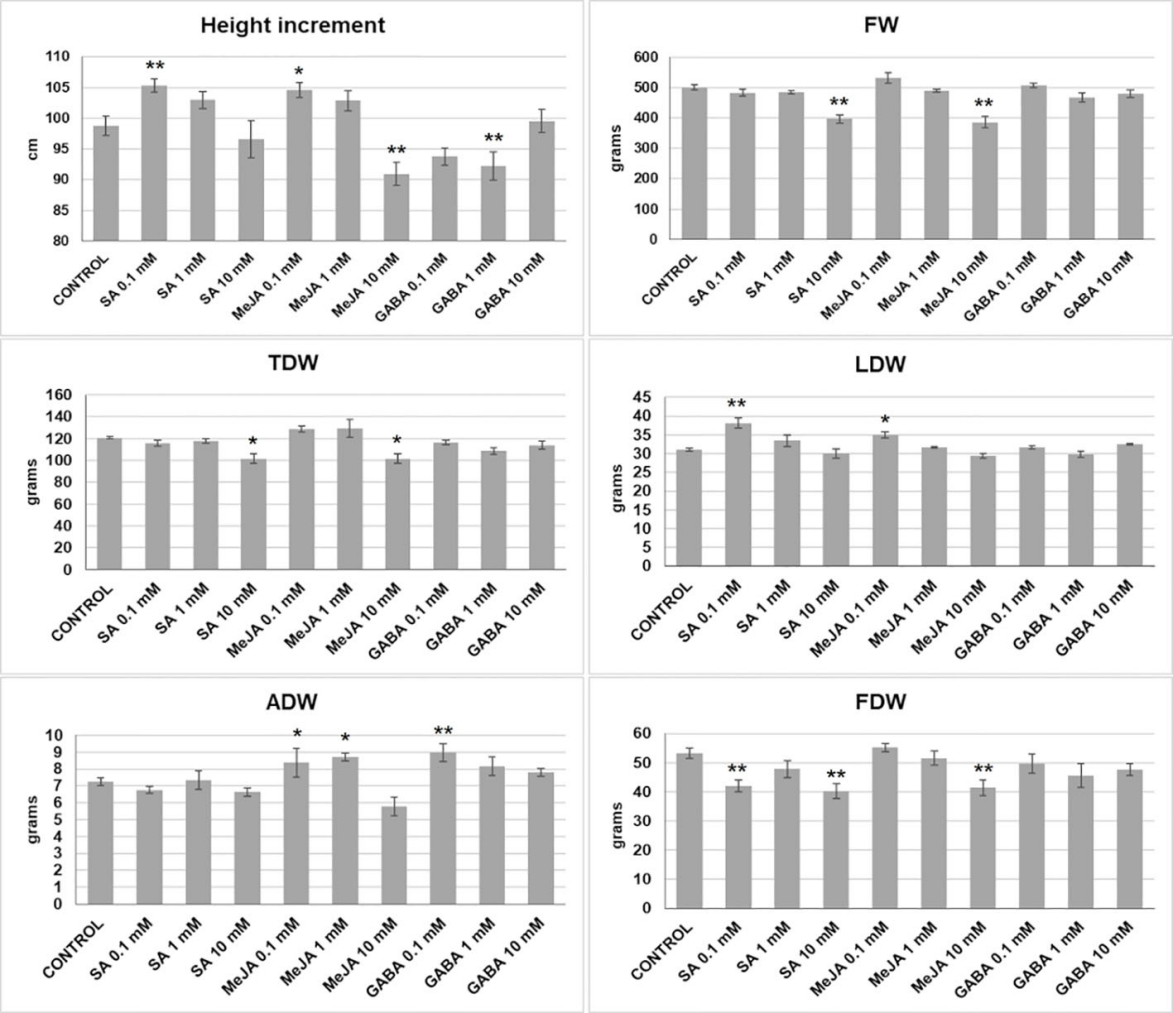
Growth parameters (increment in height, fresh and dry weights) measured in plants subjected to different treatments: fresh weight (FW), total dry weight (TDW), leaves dry weight (LDW), apical inflorescence dry weight (ADW), and total inflorescences dry weight (FDW). Mean ± standard error is represented. Asterisks indicate significant differences with control plants (one-way ANOVA test followed, by two-tailed Dunnett’s test, **p < 0.05, *p < 0.1).

Several treatments influenced plant growth and development (**Figure 1**). Low doses (0.1 mM) of SA and MeJA produced taller plants and more LDW than the control. The 0.1-1 mM doses of MeJA also increased significantly the ADW. In contrast, the highest dose (10 mM) of SA or MeJA had net negative effects on the height increment, FW, TDW, and FDW, being the last one also negatively affected by 0.1 mM SA. GABA treatments had significant effects on plant height, producing small plants when used at 1 mM, and ADW at 0.1 mM.

#### 3.1.2 Number of inflorescences and glandular trichomes density

**Figure 2:**
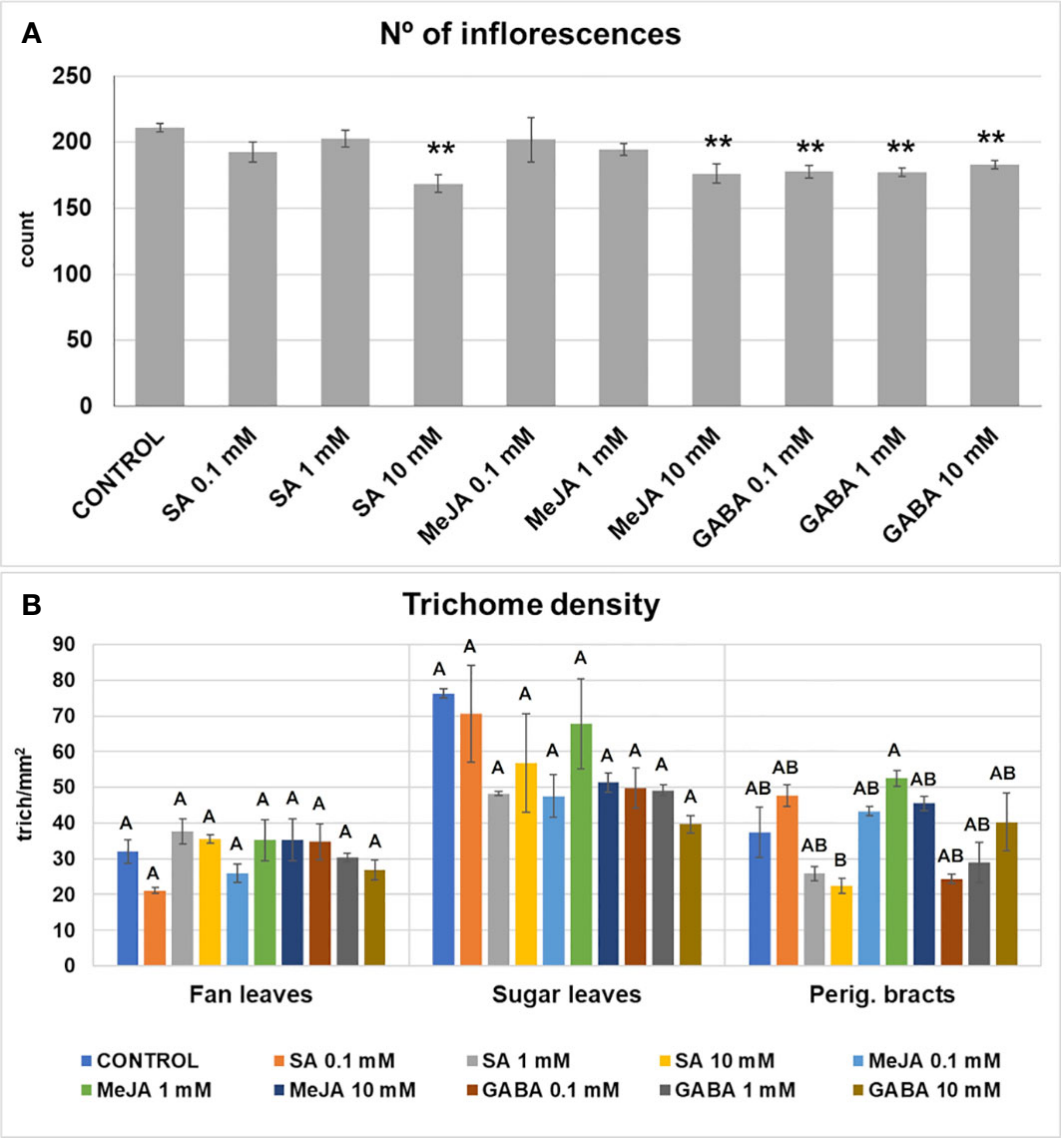
Number of inflorescences (floral buds) **(A)**, and trichome density (number of trichomes per mm^2^) **(B)** in fan leaves, sugar leaves, and perigonal bracts developed by plants subjected to the different treatments. Mean ± standard error is represented. For the number of inflorescences, asterisks indicate significant differences with control plants (one-way ANOVA test followed, by two-tailed Dunnett’s test, **p < 0.05). For the trichome density, means not sharing any letter are significantly different (K-W and Dunn’s test, p < 0.1).

Differences in the number of inflorescences were found between treatments, ranging from a minimum of 162 for the 10 mM SA treatment to 217 for the control treatment. However, significant differences with the control plants were found only in the GABA treatments, 10 mM SA and 10 mM MeJA treatments, in which the number of inflorescences decreased up to 30% (**Figure 2A**).

Major differences in trichome density in perigonal bracts were found only between the 10 mM SA and the 1 mM MeJA treatments, but not when compared with the control group, indicating that glandular trichome formation was not affected by treatments (**Figure 2B**).

### 3.2 Effects on the THCA and CBDA production

**Figure 3:**
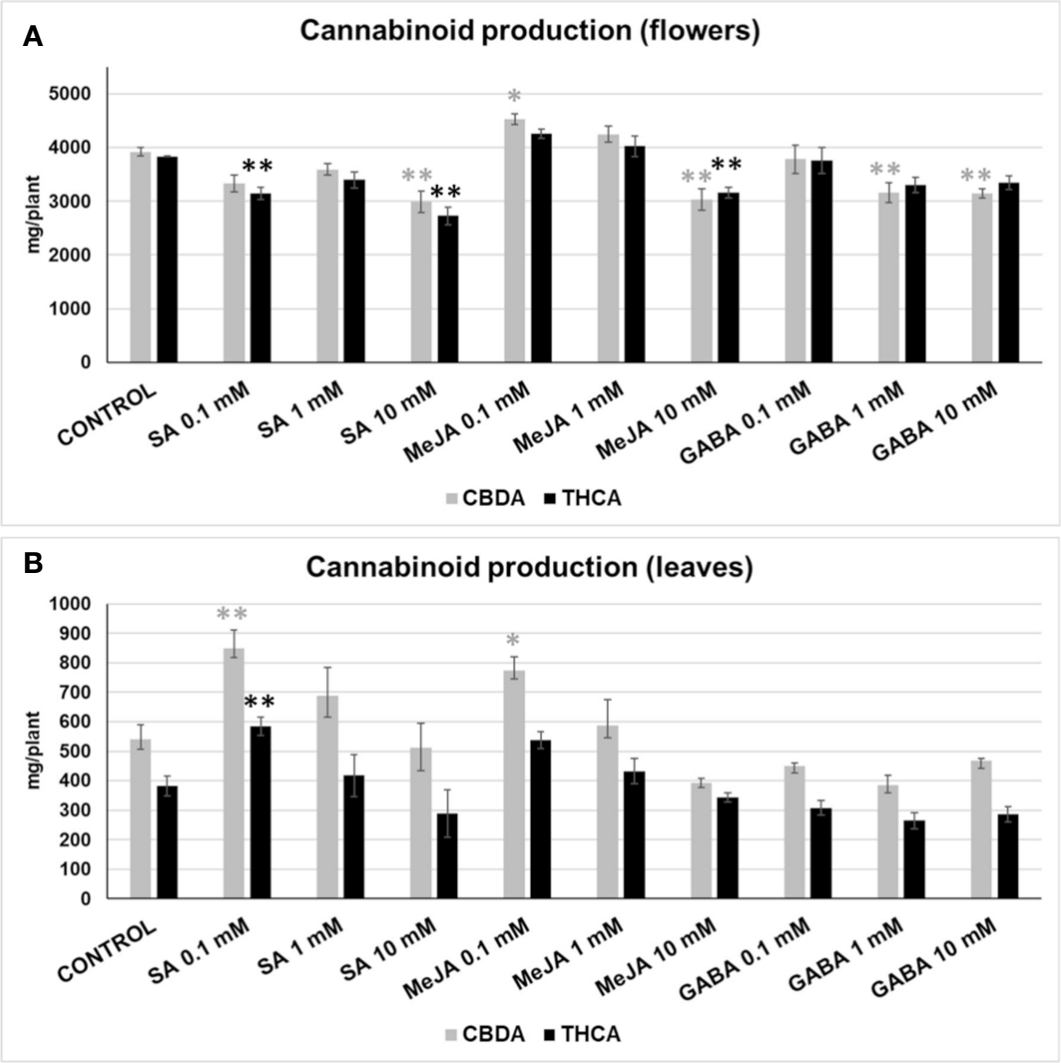
CBDA (grey) and THCA (black) produced in inflorescences **(A)**, and leaves **(B)** by plants subjected to different treatments, expressed in mg per plant. Mean ± standard error is represented. Within each cannabinoid, asterisks indicate significant differences with control plants (one-way ANOVA test, followed by two-tailed Dunnett’s test, **p < 0.05, *p < 0.1).

The effect of SA, MeJA, and GABA on the main cannabinoids (THCA and CBDA) was analysed in flowers and leaves. Some treatments affected the production of cannabinoids in medical cannabis inflorescences (**Figure 3A**). The production of CBDA was significantly increased (15.6% more than the mock) in 0.1 mM MeJA treatment. The application of 1 mM MeJA produced a smaller CBDA increase (8.42%) but was not significantly different when compared with the control. In contrast, 10 mM SA, 10 mM MeJA, and 1 mM or 10 mM GABA produced a significant reduction in CBDA yields (23.71%, 22.61%, 19.36%, and 19.8%, respectively). The THCA content was reduced significantly in 0.1 mM SA, 10 mM SA, and 10 mM MeJA treatments (17.93%, 28.87%, and 17.62%, respectively).

The exogenous application of the phytohormones SA and MeJA also had interesting effects on the production of cannabinoids in the fan leaves (**Figure 3B**). We observed a significant and high CBDA accumulation in the leaves of plants treated with 0.1 mM SA and 0.1 mM MeJA, which corresponded to an increase of 57% and 43% regarding control plants, respectively. THCA accumulation was higher in plants treated with 0.1 mM SA and 0.1 mM MeJA than in control plants by 53% and 40%, respectively, although the difference was significant only for the former.

### 3.3 Relative expression profiles of biosynthetic genes

**Figure 4:**
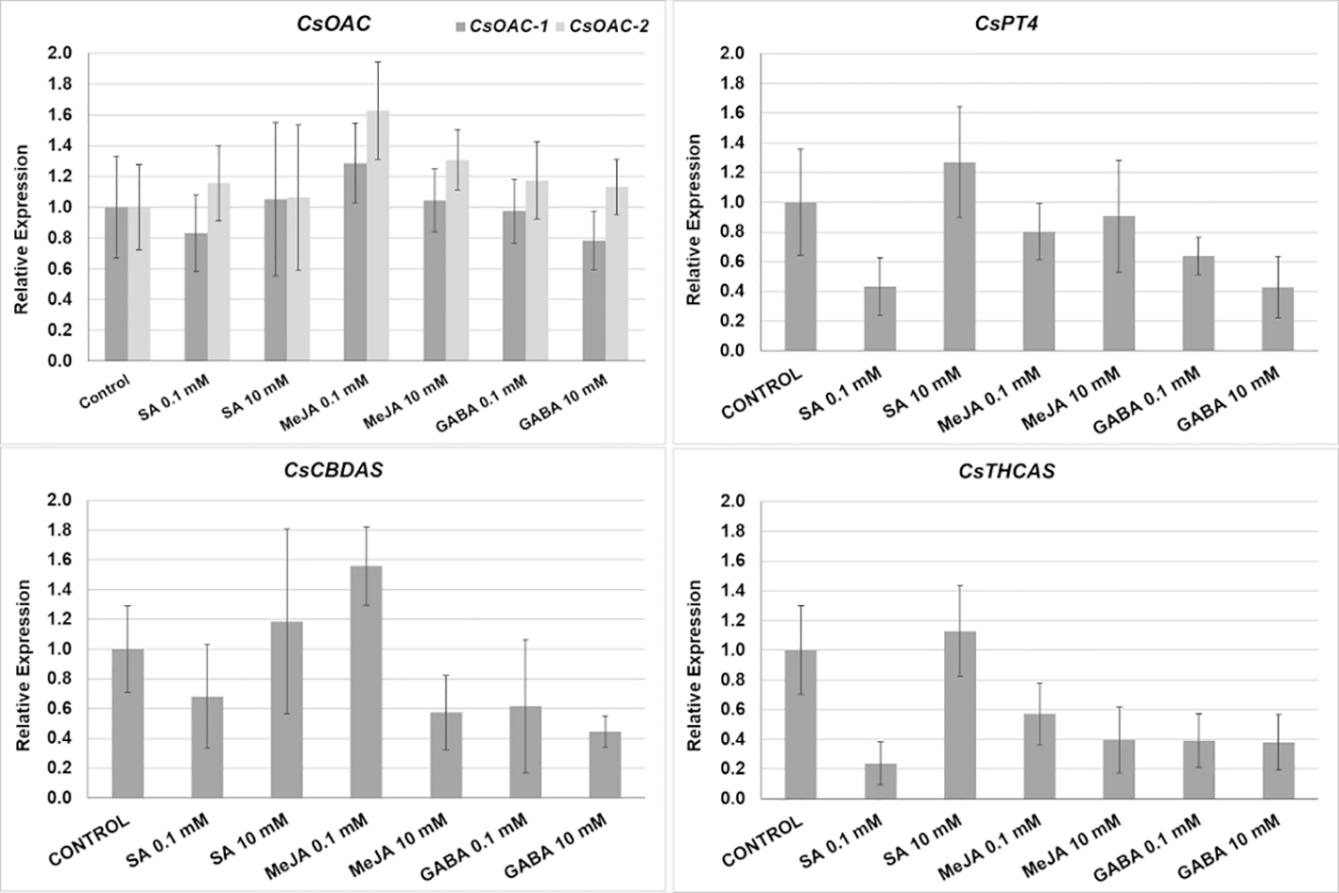
Relative expression (fold change) of cannabinoid biosynthesis pathway in plants subjected to different treatments: *CsAOC-1*, and *CsAOC-2*, *olivetolic acid cyclases -1* and *-2*; *CsPT4*, *prenyltransferase-4*; *CsCBDAS* and *CsTHCAS*, *cannabidiolic acid*, and *Δ^9^-tetrahydrocannabinolic acid synthase*, respectively. Mean ± standard error is represented. Asterisks indicate significant differences with control plants (one-way ANOVA test, followed by two-tailed Dunnett’s test, p < 0.1).

Changes in the relative expression of the genes involved in the final steps of the cannabinoid biosynthesis pathway (*CsAOC-1*, *CsAOC-2*, *CsPT4*, *CsTHCAS*, and *CsCBDAS*) were studied to assess the influence of the treatments on the gene expression. We monitored the changes caused by the application of the lowest (0.1 mM) and highest (10 mM) doses of SA, MeJA, and GABA, chosen because most of the significant changes in the THCA and CBDA production were detected in such treatments (**Figure 3A**). However, the changes measured in the relative gene expression were not significant regarding control plants (**Figure 4**).

### 3.4 Correlation analysis of plant growth, glandular trichome density and cannabinoid yield

**Figure 5:**
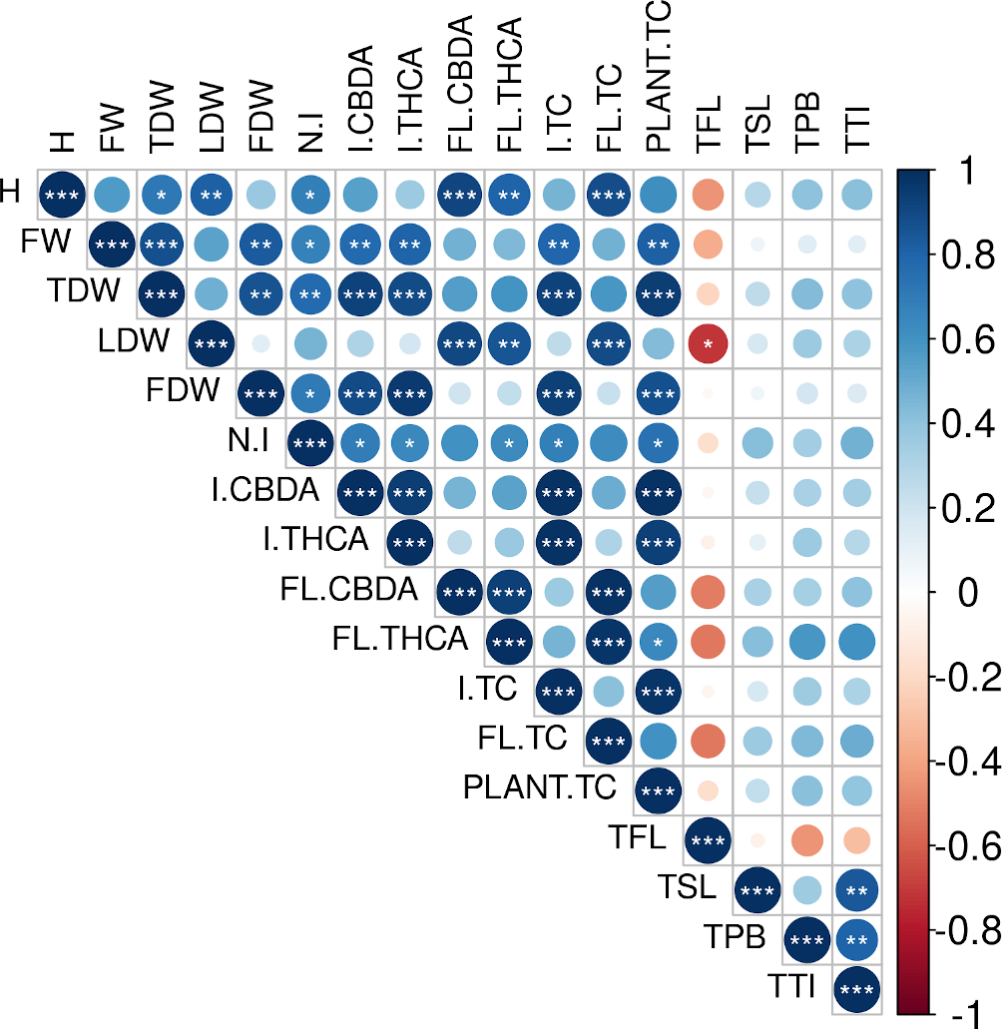
Correlogram depicting Pearson´s correlation matrices between plant growth parameters and cannabinoid yields. Correlation coefficients are proportional to the dot area and color. Correlation p-value is indicated by asterisks (*, p < 0.05; **, p < 0.01; ***, p < 0.001). H, height increment; FW, fresh weight; TDW, total dry weight; LDW, leaves dry weight; FDW, total inflorescences dry weight; N.I, total number of inflorescences; I.CBDA, CBDA content in inflorescences; I.THCA, THCA content in inflorescences; FL.CBDA, CBDA content in fan leaves; FL.THCA, THCA content in fan leaves; I.TC, total cannabinoid content in inflorescences; FL.TC, total cannabinoid content in fan leaves; Plant.TC, plant total cannabinoid content; TFL, trichome density in fan leaves; TSL, trichome density in sugar leaves; TPB, trichome density in perigonal bracts; TTI, total trichome density in inflorescences.

Some treatments seem to influence both, plant growth and cannabinoid yields (see **Figures 1**, **3**). To investigate the possible relation between both phenomena, we calculated Pearson’s correlations between the plant growth parameters described above (height increase, FW, TDW, LDW, FDW, and the number of inflorescences); trichome densities in fan leaves, perigonal bracts, sugar leaves, and total trichomes in inflorescences (sum of trichome density in perigonal bracts, and sugar leaves), and the cannabinoid content in leaves, inflorescences, and the plant total (**Figure 5** and **Supplementary Table 2**). Results show strong correlations between plant growth and cannabinoid accumulation. For example, total produced cannabinoids are strongly correlated with growth parameters such as FW, TDW, and FDW, while the cannabinoid production in inflorescences and leaves correlated with the height increment, the number of inflorescences, FDW, and LDW. In contrast, correlations between the trichome density in both inflorescences and leaves and the production of cannabinoids were non-significant.

## 4 Discussion

### 4.1 Effect of the signaling compounds SA, MeJA and GABA on plant development

Elicitor molecules, phytohormones, and their synthetic analogs have been successfully used in many plant species to trigger resistance mechanisms against pathogens and enhance the synthesis of secondary metabolites. Particularly, SA, JA, and their analogs can trigger regulatory changes in the SA- or JA-mediated defense pathways and have been widely used to enhance the synthesis of alkaloids, terpenoids, and other products in plant tissues and cells (Bektas and Eulgem, 2015; Palmer et al., 2019; Jan et al., 2021). However, the modification of the hormonal balance may have fitness costs, affecting the plant development and causing undesirable side effects such as dwarfism, delayed flowering, and reduced seed production (Denancé et al., 2013; Huot et al., 2014; Vos et al., 2015). Therefore, the application of phytohormones and other plant signaling molecules must be carried out carefully. Plants used in this experiment grew healthy and didn’t show any noticeable alteration, except for plants treated with 10 mM SA, in which some leaves started showing burnt tips and margins two or three weeks before harvesting. Nevertheless, we have detected negative effects on height increment, FW, TDW, and FDW predominantly in doses of SA and MeJA higher than 0.1 mM, whereas at their lowest concentration (0.1 mM), plants showed significant height and LDW gains. On the other hand, GABA produced little changes in plant growth, except for a height diminution similar to the 10 mM MeJA treatment (significant for 1 mM GABA), a generalized decrease in the number of inflorescences, and a significant increase in ADW (up to 23.87% regarding the control) with 0.1 mM GABA.

Several phytohormones as SA or JA among others (such as GA_3_, ABA, brassinosteroids, cytokinins, auxins, and ET) are flowering regulators (Conti, 2017) influencing the initiation, growth, and development of plant trichomes by regulating the expression of several gene families (Li et al., 2021a; Wang et al., 2021). The general ability of phytohormones to alter the trichome initiation is conserved across angiosperms, but the particular regulation mechanisms and the formation of the different trichome types seem different among plant species (Maes and Goossens, 2010; Wang et al., 2021), which could explain why we have not detected substantial changes in glandular trichome density elicited by SA or MeJA treatments in the 0.1-10 mM range. Regarding GABA and to the best of our knowledge, its involvement in trichome biology has not been studied. Our results indicate that this compound doesn’t influence trichome formation in cannabis. These results also point out that, in cannabis, these three signaling compounds are not tightly regulating trichome formation. Besides, we could not detect clear stimulatory or inhibitory effects of SA MeJA, and GABA on the inflorescence development, although principally the higher doses of SA and MeJA had negative effects on the FDW and the number of inflorescences, whereas GABA treatments decreased the number of inflorescences. However, we cannot dismiss potential effects on the total number of phytomers and individual flowers developed within an inflorescence, which could be elucidated in future experiments. Other signaling compounds not tested in this study, such as gibberellins, cytokinins, auxins, ET, and ABA, could produce changes in the flowering behavior and trichome development or in the regulation of the cannabinoid biosynthetic pathway. In fact, changes in cannabinoid profiles have been reported in cannabis after treatments with ABA (Mansouri et al., 2009b), GA_3_ (Mansouri et al., 2009a), and ethephon (Mansouri et al., 2016).

So far, the effects of the exogenous application of SA, MeJA, and GABA on the growth of medical cannabis plants have not been reported. We have found that moderate doses of SA and MeJA are harmless for plants and even stimulate height growth and LDW, in contrast with the highest doses assayed, which affected negatively mainly height increment, FW, TDW, and FDW. However, substantial effects on inflorescence and trichome formation were not detected.

### 4.2 Low doses of MeJA increase the cannabinoid yield in inflorescences

Cannabis female inflorescences are the richest plant source of the valuable cannabinoids, produced and stored by the stalked glandular trichomes of the florets and sugar leaves. Thus, floral buds are the preferred plant material for the recreational and medical cannabis industry. As shown in **Figure 3A**, low doses of MeJA were the only ones producing an increase in the CBDA, reaching up to 15.6% more content than control plants for the 0.1 mM MeJA treatment. In addition, the 1 mM treated plants show an increased CBDA content of 8.42%, but not significantly different from the control plants. The THCA yields also increased on the inflorescences developed in the 0.1-1 mM MeJA treated plants by 11% and 5%, respectively, although these differences were not significantly different from the control plants. Therefore, low doses of MeJA, particularly 0.1 mM, are effective in increasing the cannabinoid yield in the inflorescences of medical cannabis. A recent study (Apicella et al., 2022) also reported an increase of the THC content in inflorescences of a marijuana variety (“White Tangy Haze”) sprayed with 0.1-1 mM MeJA, suggesting that this treatment can be species-wide used to enhance cannabinoid yields in *Cannabis sativa*. On the other hand, our results using SA and GABA contrast with those obtained by Jalali et al. (2019), indicating either that the elicitation effects of exogenously delivered signaling molecules could be affected by the lack of genetic uniformity in the seed-derived population, environmental conditions, or that they could be genotype-dependent to some extent (Schwabe and McGlaughlin, 2019; Toth et al., 2020; Johnson and Wallace, 2021).

### 4.3 Low doses of SA and MeJA increase the cannabinoid yield in leaves

The cannabis leaves are a minor source of cannabinoids compared with the plant inflorescences. However, leaf biomass represents a significant portion of the whole plant (around 30% of TDW), containing relatively high quantities of cannabinoids in medical cannabis varieties, and is potentially worth as a source for the obtention of extracts and purified compounds for the pharmaceutical industry. Leaves collected from 0.1 mM SA and 0.1 mM MeJA treated plants increased significantly their CBDA yield by 57.31% and 43.18% to control plants, respectively. Also, the THCA yield was increased by as much as 52.68% in the 0.1 mM SA treatment (significant), and by 40.27% in the 0.1 mM MeJA treatment (non-significant) (**Figure 3B**). Interestingly, this result invites speculation about if the cannabinoid biosynthesis in leaves could be regulated differently than in inflorescences. Nevertheless, both the 0.1 mM SA and MeJA treatments produced a sharp increase in the cannabinoids yield in medical cannabis leaves, improving the profitability of this plant material as a source of cannabinoids.

### 4.4 Cannabinoid yield does correlate well with plant growth, but not with trichome density or expression of final biosynthetic genes

The analysis of the possible correlations between several growth parameters and cannabinoid yields shows that cannabinoid production is strongly correlated with most of the quantified growth parameters. However, a correlation between the trichome density and the production of cannabinoids was not found, contrastingly with reports in e.g., tomato (Hua et al., 2020), *Artemisia annua* (Maes et al., 2011) or peppermint (Cappellari et al., 2019), in which an increase of terpenoids was accompanied by higher glandular trichome densities. On the other hand, the expression of *CsAOC-1*, *CsAOC-2*, *CsPT4*, *CsTHCAS*, and *CsCBDAS* genes didn´t experience significant changes after the application of treatments, indicating that they reacted in a time window smaller than 24 hours or that they don’t affect at all their expression. However, several putative *cis-*regulatory elements for hormone-responsive transcription factors, like the MYB and WRKY families, have been described at least in the promoter regions of the *CsTHCAS* and *CsCBDAS* (Singh et al., 2021), which could potentially regulate their expression in response to hormonal changes. Therefore, it is likely that the expression of other biosynthetic genes could be modulated, increasing the amounts of precursor molecules used for cannabinoid biosynthesis. For example, MeJA treatments induced the up-regulation of the *geranylgeranyl diphosphate synthase* genes in cotton (Ali et al., 2020), *Catharanthus roseus* (Rai et al., 2013), and *Litsea cubeba* (Zhao et al., 2020), enhancing the contents of terpenoids and biomass, and could potentially increase the biosynthesis of the cannabinoid precursor CBGA in medical cannabis.

It is known that the exogenous applications of salicylates and jasmonates can affect plant growth and crop productivity, depending on the plant species and the promoting or inhibitory effects of concentrations used, particularly for SA (Rivas-San Vicente and Plasencia, 2011; Li et al., 2022). For example, JA and MeJA improved growth and photosynthetic activity in *Brassica oleracea* seedlings (Sirhindi et al., 2020). Foliar sprays of SA and MeJA stimulated the accumulation of essential oils in peppermint in a dose-dependent manner, although higher doses of MeJA inhibited plant growth (Cappellari et al., 2019). SA application in *Achillea millefolium* resulted in linear increases in biomass accumulation of roots, total dry mass, and chlorophylls and elicited the production of essential oils and total phenols (Gorni and Pacheco, 2016). Besides, SA and its derivatives applied as a foliar spray to pomegranate improved crop yield and the production of phenolics, anthocyanins, and ascorbic acid (García-Pastor et al., 2020).

Therefore, we propose that the measured effects of these stress-related signaling compounds on the cannabis plants were caused by metabolic tradeoffs influencing the plant’s fitness, and changes in cannabinoid accumulation were a result of the modulation of multiple biosynthetic pathways, which are also related to the plant’s developmental responses.

## 5 Conclusions

We have found that small doses of the phytohormones SA and MeJA can enhance growth and biomass production of medical cannabis and lead to significant changes in the accumulation of CBDA and THCA in leaves and inflorescences, depending on the compound and concentration used. Remarkably, 0.1 mM MeJA was the most beneficial treatment to increase the CBDA content in inflorescences, while SA and MeJA at 0.1 mM increased the THCA and CBDA accumulation in both inflorescences and leaves. We propose that the application of 0.1 mM MeJA during the flowering stage can be used as a booster of CBDA accumulation in inflorescences of medical cannabis, whereas 0.1 mM SA and 0.1 mM MeJA is suitable for increasing the CBDA and THCA yields in leaves.

More research is needed to understand the precise effects of several signaling compounds on the intricate regulation of cannabinoid biosynthesis and the physiology of the medical cannabis varieties. It is also possible that the application of other elicitor substances related to the SA- and JA-pathways, such as Benzothiadiazole (BTH), chitosan, or hexanoic acid, among others, could produce similar results. Moreover, the use of such substances in the production of medical cannabis could also be beneficial for the pathogen and pest control in this valuable crop, in addition to enhancing cannabinoid yields.

## Data availability statement

The raw data supporting the conclusions of this article will be made available by the authors upon reasonable request.

## Author contributions

JG and CF-V conceived the study. JG, CC and SR conducted the main experiments and analysed data. CF-V, CS and NV collaborated in funding acquisition and provided resources. JG wrote the original draft. JG, CC, SR, NV, CS, JM-Q and CF-V collaborated in review and editing. All authors contributed to the article and approved the final version. 
